# Studies of Ion Kinetic-Energy Distributions in the Gaseous Electronics Conference RF Reference Cell

**DOI:** 10.6028/jres.100.029

**Published:** 1995

**Authors:** J. K. Olthoff, R. J. Van Brunt, S. B. Radovanov

**Affiliations:** National Institute of Standards and Technology, Gaithersburg, MD 20899-0001

**Keywords:** argon-oxygen plasmas, ion bombardment, ion energy analyzers, ion kinetic-energy distributions, mass spectrometry, radio-frequency discharges

## Abstract

A review is presented of kinetic-energy distribution measurements for ions striking grounded surfaces in a Gaseous Electronics Conference (GEC) rf Reference Cell. Two experimental arrangements that have been used to measure ion energies in the GEC Cell are described, and a comparison of their performance under different operating conditions is presented. Significant results from ion-energy analysis in the Reference Cell are highlighted, including evidence of effects due to surface conditions on ion sampling, verification of electrical behavior of the cell, inferences about ion-molecule reactions indicated by the shapes of measured ion kinetic-energy distributions (IEDs), and the use of measured IEDs for the validation of theoretical models. The paper concludes with a detailed study of IEDs measured for rf plasmas generated in mixtures of argon and oxygen, using both experimental arrangements.

## 1. Introduction

Ion bombardment plays a key role in the plasma etching of semiconductor materials [1], and therefore determination of the identity, flux, and energies of ions in plasma reactors has attracted much interest. Davis and Vanderslice [2] performed one of the earliest investigations of the identity and energy of ions striking the electrodes in a dc glow discharge by sampling ions through a small hole in the cathode, and then determining their distribution of kinetic energies with an electrostatic analyzer. Coburn and Kay [3] applied this technique to radio-frequency (rf) discharges in 1972, and then proceeded to extend the use of the technique to measure rf plasma potentials [4].

Subsequently, the measurement of ion kinetic-energy distributions (IEDs) in low pressure, rf discharges generated in parallel-plate reactors has been used to investigate: (1) ion bombardment of the grounded electrode in argon plasmas [5–10]; (2) the role of ions in discharges sustained in etching gases [11–13]; (3) the angular distribution of ions striking the electrodes [14,15]; and (4) the effect of symmetric charge-transfer collisions on the shapes of IEDs [16]. Additional experiments have been performed to measure the energies of ions striking the surface of the powered electrode in capacitively coupled rf discharges [13,15, 17–19].

The first measurements of ion kinetic energies in the GEC rf Reference Cell were performed for rf discharges in argon. Two methods of sampling ions from the discharge were used: (1) through a 0.2 mm orifice in the face of a stainless-steel cone inserted into the side of the rf glow between the parallel-plate electrodes [20], and (2) through a 0.1 mm orifice in the grounded, parallel-plate electrode [21]. Subsequent experiments measured the IEDs at the grounded electrode of rf discharges sustained in helium, oxygen, nitrogen [22], and hydrogen [23]. Ions sampled from rf discharges sustained in mixtures of gases, such as Ar-He [24], Ar-H_2_ [25], and He-N_2_ [26] have also been studied, and preliminary results have been reported for plasmas generated in mixtures of argon with oxygen [27] and nitrogen [28].

In this paper the two methods that have been used to measure IEDs in the Reference Cell are described, and the applications and interpretations of ion-energy measurements reported in the literature for GEC Cells are reviewed. The paper concludes with a detailed study of IEDs measured from discharges generated in argon-oxygen mixtures in the GEC Cell, both at the grounded electrode and at the side of the discharge. The differences and similarities between the sampling techniques and the instruments are analyzed.

## 2. Experiments

All of the research on ion-energy distributions in the GEC rf Reference Cell has been performed in a single GEC Cell installed at the National Institute of Standards and Technology. This cell is essentially a normally configured GEC Cell [29] with the lower electrode powered by a 13.56 MHz power supply through a 0.1 μF blocking capacitor and an rf filter box [30]. A shunt circuit [29] is used to minimize the displacement current, and the current and voltage waveforms, including the applied rf peak-to-peak voltage (*V*_pp_), are measured at the base of the powered electrode by a digital oscilloscope. An equivalent circuit of the cell is used to determine the voltage and current waveforms at the surface of the powered electrode exposed to the discharge [29,31], and the power dissipated in the plasma. Modifications were made to accommodate the different ion energy analyzer-mass spectrometer systems discussed below.

**Fig. 1:**
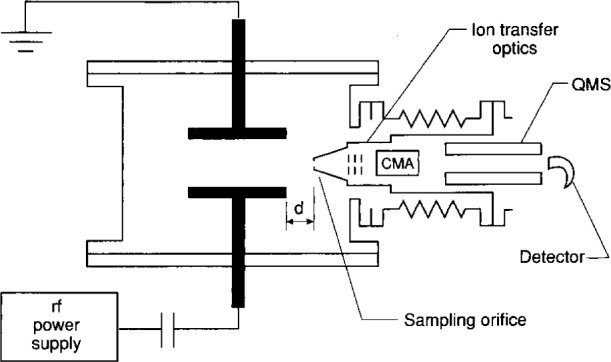
Schematic diagram from Ref. [20] of the VG SXP300H/CMA mass spectrometer-ion energy analyzer attached to the GEC rf Reference Cell. The variable *d* is the distance from the face of the sampling cone to the edge of the electrodes, and may be varied from 0 cm to 10 cm. CMA is the cylindrical mirror analyzer, and QMS is the quadrupole mass spectrometer.

The first ion kinetic-energy measurements reported in the GEC rf Reference Cell [20] were obtained using a Vacuum Generator SXP300H/CMA^1^ mass spectrometer (MS) equipped with a cylindrical mirror ion-energy analyzer (CMA) [32]. A schematic diagram of the experimental apparatus is shown in Fig. 1. The CMA/MS system mounts via a bellows assembly to the 6-in port on the GEC Cell that is opposite the turbo pumping port. Ions are sampled through a 0.2 mm aperture in the face of a grounded, stainless-steel sampling cone which is positioned at the side of the discharge at the mid-plane between the parallel electrodes. The distance from the edge of the electrodes to the cone face can be varied from 0 cm to 10 cm. Ion kinetic-energy distributions are acquired by tuning the mass spectrometer to a particular mass-to-charge ratio, and then scanning the energy of the ions entering the CMA. The full-width-at-half-maximum (FWHM) energy resolution of the energy analyzer was normally set to 0.5 eV over the energy range used for this instrument [20,27].

One advantage of this experimental arrangement is the ease of installation on the GEC Cell. The mass spectrometer system is simply mounted to the 6-in con-flat flange, and no further modification to the cell is required. An additional advantage is the ability to adjust the location of the sampling orifice with respect to the plasma, thus providing a mechanism for investigating ion-molecule reactions within the discharge while also allowing a means of removing the sampling cone from the discharge region. While the presence of the probe affects the local characteristics of the plasma near the probe face, the bulk characteristics of the plasma, as determined by electrical and optical measurements, exhibit only minor changes when the probe is inserted into the discharge. Studies have shown that IEDs measured with the surface of the sampling cone in close proximity to the glow discharge exhibit many of the same characteristics as IEDs measured through an aperture in the grounded electrode [21]. This is expected because a sheath forms between the face of the grounded sampling cone and the glow of the discharge, as happens in front of the grounded parallel-plate electrode. However, if the edge of the sheath region, or “dark space,” in front of the powered electrode becomes large enough to approach the orifice in the sampling cone, as can happen at low pressures (< 7 Pa in Ar), then the sheath exhibits a strong two-dimensional nature, and few ions are accelerated into the aperture with the appropriate trajectories for detection [20]. This limits the range of plasma conditions that can be investigated using an ion sampling probe inserted into the “side” of the plasma.

**Fig. 2:**
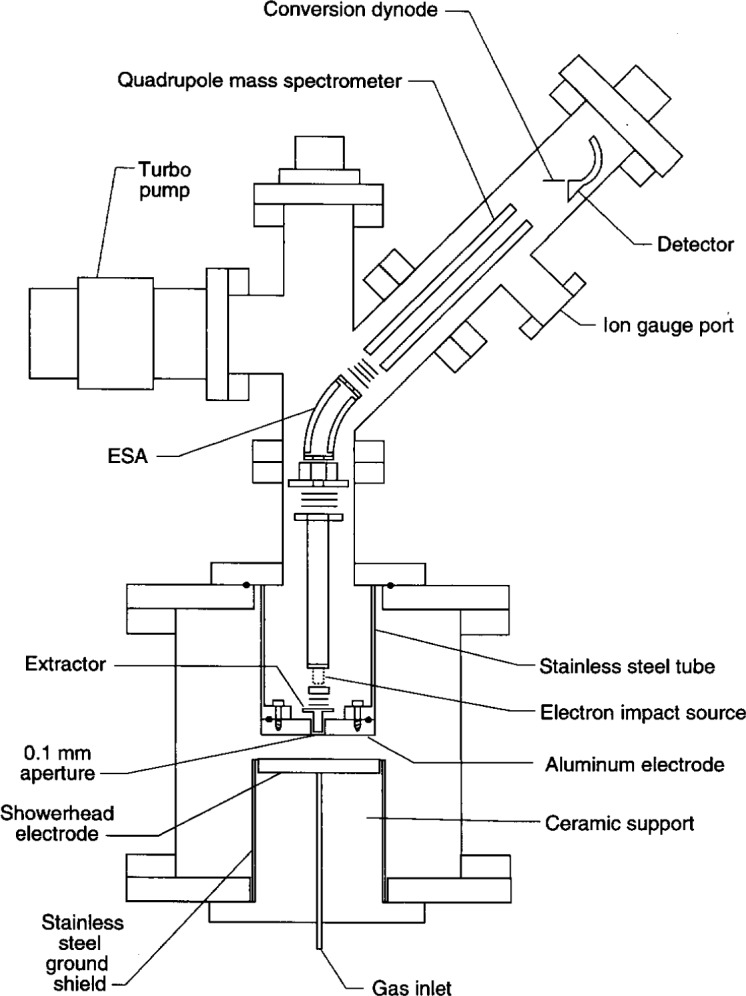
Schematic diagram from Ref. [21] of the Hiden EQP Plasma Probe mass spectrometer-ion energy analyzer attached to the GEC rf Reference Cell. The ESA is a 45° electrostatic ion-energy analyzer.

A second experimental set-up used for extensive investigations of IEDs in the GEC Cell [21–26,28] is shown in Fig. 2. In this apparatus, ions are sampled through an 0.1 mm aperture in the upper (grounded) electrode of the cell, and the mass and energy are analyzed by a Hiden EQP Plasma Probe. In order to sample ions through the electrode, it was necessary to move the showerhead electrode of the GEC Cell (normally in the upper position) to the lower electrode position, and to redesign the upper electrode assembly to accommodate the EQP system as shown in the figure. The ceramic insulator on which the upper electrode is normally mounted was replaced with a stainless-steel tube to which the grounded electrode containing the aperture was attached. No additional ground shield is required since the entire support assembly is made of stainless steel, and no water cooling capabilities are incorporated in the design because of the low plasma powers used (< 10 W). Measurements of the electrical waveforms indicate that the modification of the grounded electrode assembly does not significantly affect the behavior of a discharge in the cell [21]. Ion-energy distributions are measured with the EQP apparatus in a manner similar to that described for the CMA/MS system. The mass spectrometer is set to a particular charge-to-mass ratio, and the energy of the ions entering the electrostatic energy analyzer (ESA) is scanned.

**Fig. 3:**
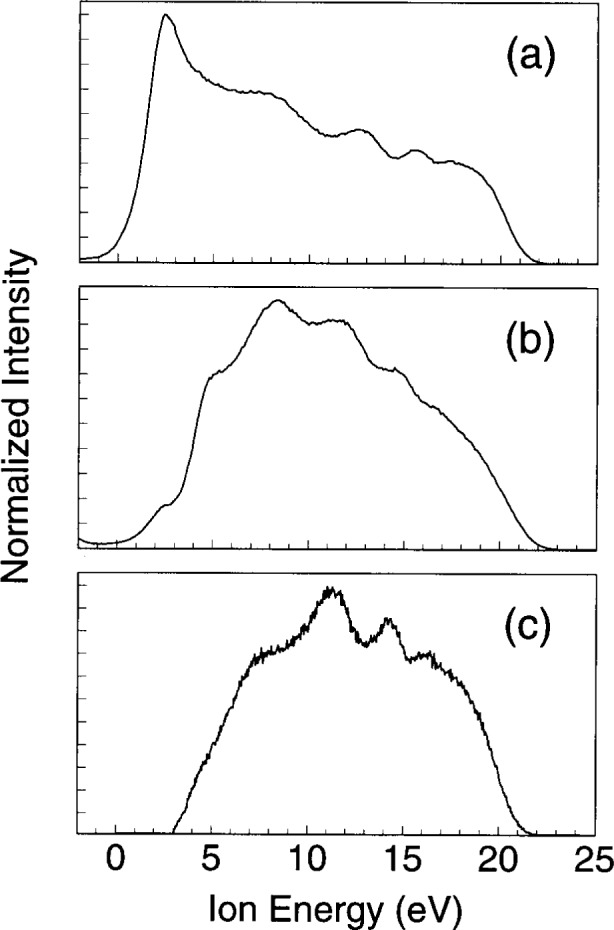
Kinetic-energy distributions of Ar^+^ ions sampled from 13.3 Pa argon discharges at *V*_pp_ = 200 V under the following experimental conditions: (a) Ions sampled through an aperture in a clean stainless-steel grounded electrode and analyzed by the EQP analyzer; (b) Ions sampled through an aperture in an aluminum electrode and analyzed by the EQP analyzer; and (c) Ions sampled through an aperture in a stainless-steel sampling cone (surface conditions unknown) and analyzed by the CMA/MS analyzer.

Unlike the CMA, which has an axial configuration [32], the ESA deflects the energy-selected ions through a fixed 45° angle (see Fig. 2). This and other differences between the two instruments are itemized in Table 1. As discussed later, the shapes of the IEDs, measured with both systems, have been shown to be very similar for argon plasmas [21], despite these differences (see Figs. 3b and 3c).

Of the parameters affecting the measurement of IEDs, the most critical and least understood is that attributed to the condition of the surface through which the ions are sampled, in particular, the effect of surface charge in the region near the orifice. The presence of surface charge near the sampling orifice can cause time (phase) dependent shifts in the ion energy scale [20,33], and/or discrimination against the detection of ions with low energies (< 10 eV) [33,34], as discussed below. Recent studies [33] have shown that the sampling of ions through an aperture in a clean stainless-steel surface (rather than an aluminum surface or a surface coated with a layer of sputtered material) yields measured IEDs that are not significantly distorted by either low-energy discrimination or energy-scale shifts. This observation is reflected in Fig. 3, which shows three IEDs obtained for ostensibly identical argon plasmas under three different ion sampling conditions: (a) ions sampled through a clean stainless-steel electrode and analyzed with the EQP system; (b) ions sampled through an aluminum electrode and analyzed with the EQP system; and (c) ions sampled through a stainless-steel cone and analyzed with the CMA/MS system.

In Fig. 3a the ion-energy distribution extends from slightly below 0 eV up to 23 eV, with a maximum intensity observed near 2 eV (the details of the shape and structure of the IED will be discussed later). This is in reasonable agreement with experimental studies utilizing retarding potential analyzers [10,14] and Monte Carlo calculations [7,14], that indicate that the ion signal under the “collisional” plasma conditions used here should increase in intensity down to near 0 eV due to charge-exchange collisions in the sheath. However, if the ions are sampled through an orifice in an aluminum electrode (Fig. 3b), a significant portion of the low energy ions is not detected, resulting in the energy distribution peaking near 8 eV. While the exact cause of this discrimination is unknown, it is thought to result from the defocussing of the sampled ions outside of the acceptance angle of the ion-energy analyzer by the presence of surface charge near the sampling orifice [33]. For the IED obtained under the CMA/MS configuration (Fig. 3c), the ion signal peaks near 11 eV, and no signal is detected at energies below approximately 3 eV. Studies of the performance of the CMA/MS apparatus on a constant-field drift tube [35] and in low-current, dc (Townsend) discharges [34] have suggested that the lack of ion signal below 3 eV is likely due to a shift in the entire energy scale that is caused by localized surface charging on the sampling cone that accelerates the ions prior to entering the analyzer. Previously published IEDs obtained with this experimental configuration have been modified to account for this energy shift [20,27,34], so that they more accurately reflect the actual kinetic energies of the ions as they strike the surface of the electrode. However, it has not yet been definitively shown whether the lack of signal below 3 eV for measurements in rf discharges made with the CMA/MS system is the result of such an energy shift, or whether this instrumental arrangement exhibits significant low energy discrimination, and ions with less than 3 eV kinetic energy simply do not enter the energy-analyzer region. These questions must be considered when attempting to determine the uncertainties of the energy scales of the measured IEDs.

**Fig. 4:**
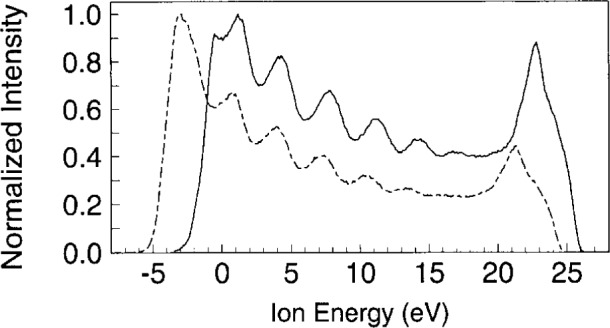
Kinetic-energy distributions from Ref. [33] of O_2_^+^ ions sampled from oxygen discharges at 8.0 Pa with stainless-steel (solid lines) and aluminum (dashed lines) grounded electrodes for *V*_pp_ = 200 V.

**Fig. 5:**
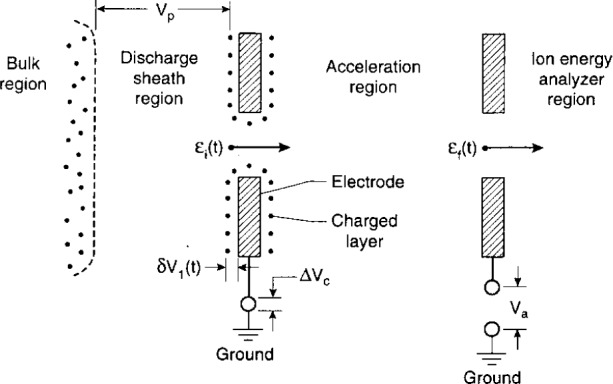
Diagram from Ref. [33] illustrating different regions of ion acceleration from the plasma glow (bulk) to the analyzer entrance aperture.

The magnitude and extent of the ion signal that extends below 0 eV in Fig. 3a is approximately that expected for the energy resolution of the EQP energy analyzer. However, significantly greater ion signals have been observed below 0 eV for certain plasmas, such as in O_2_, He, H_2_, and N_2_, when the ions are sampled through an aluminum electrode and analyzed by the EQP system [33]. An example of this effect is shown in Fig. 4 for O_2_^+^ ions sampled from 8 Pa oxygen plasmas with both stainless-steel and aluminum grounded electrodes. The IED obtained with the aluminum electrode clearly extends further into the negative energy range, apparently due to a shift of the energy scale of the IED, as compared to the IED obtained with the stainless-steel electrode. For some gas mixtures, such as 5 % SF_6_ in argon, ion signals have been detected down to −10 eV [36]. This effect may be the result of charging of the aluminum surface surrounding the sampling orifice [33], as illustrated in Fig. 5.

Figure 5 shows the possible influence on measured ion energies of electrode surface charge surrounding the sampling aperture. When ions enter the aperture they have, at some time *t* in the rf cycle, a kinetic energy *ε*_i_(*t*). After passing through the aperture, the ions are accelerated by the acceleration potential which is affected by *δV*_1_(*t*), the charged surface-to-ground potential difference at time *t* on the electrode containing the aperture. The ions that enter the energy analyzer region after acceleration then have an energy given by:

$$\varepsilon_{f}(t)=\varepsilon_{i}(t)+eV_{a}+e[\delta V_{1}(t)+\Delta V_{c}]$$ (1)

where *V*_a_ is the applied acceleration voltage and D*V*_c_ is any contact potential. The term in brackets is an error (or shift) in the ion energy recorded relative to ground potential due to surface charging and contact potentials. The direction of the shift due to surface charging is determined by the polarity of the surface charge. Ion signals can be recorded with “negative energies” if [*δV*_1_(*t*) + Δ*V*_c_] < 0.

It should be noted that in a capacitively-coupled rf discharge, the time-averaged plasma potential, *V*_p_ indicated in Fig. 5, is likely to be shifted relative to ground by the amount *δV*_1_(*t*) due to surface charge. Thus, the maximum allowed ion energy relative to ground, which is determined by *V*_p_ (approximately 25 eV in Fig. 4), will also shift by this amount, while the maximum allowed ion energy *relative to the electrode surface potential* will remain unchanged. In general, surface charge density may depend on time (rf phase), and therefore, the shift due to *δV*_1_(*t*) has a time dependence which would imply that the shift will not necessarily be the same for all ion energies [16]. Under steady-state conditions, *δV*_1_(*t*) is a periodic function with a period of one rf cycle. Additionally, the surface-charge density may not be uniform in the area surrounding the orifice, which would make the magnitude of the energy shift difficult to quantify.

As can be seen from Fig. 4, the use of a clean stainless-steel electrode appears to minimize this effect, most probably because of reduced surface charging resulting from the lack of a non- or partially-conducting oxide layer. This suggests that sampling of ions through the stainless-steel grounded electrode allows the measurement of undistorted IEDs, i.e., IEDs that more accurately reflect the distribution of ion energies as they strike the electrode surface. Experience has shown that distortions of the IEDs, similar to those observed for aluminum electrodes, are observed with the stainless-steel electrode after the clean electrode has been exposed to a discharge for several hours. This has been attributed to the sputtering of material from the aluminum powered electrode which eventually deposits a layer of aluminum oxide on the stainless-steel grounded electrode. Similar deposition has been observed in hydrogen plasmas generated in a GEC Cell with a “split” electrode [37]. This effect will be discussed further in Sec. 4 of this paper as it relates to the analysis of IEDs obtained from rf plasmas sustained in mixtures of argon and oxygen.

## 3. Review of Previous IED Investigations on the GEC Cell

Perhaps the most readily obtained and useful information to be derived from mass-selected IEDs is the identity of the dominant ion striking the grounded electrode. While much can be learned by observing the IEDs of the minor ions present in the plasma, as will be shown later, the identity and shape of the IEDs of the dominant ion are of primary interest both to the experimentalist and to the theorist attempting to model the discharge. For most of the plasmas for which IEDs have been measured in the GEC Cell, the dominant ion is the parent ion of the feed gas (e.g., Ar^+^ in argon, N_2_^+^ in nitrogen), with minor ions contributing less than 10 % to the total ion current. This has been shown for discharges sustained in argon [20,21], oxygen, nitrogen, and helium [22] under a wide range of pressures and applied voltages. Studies performed in pure hydrogen [23], however, show that either H_2_^+^ or H_3_^+^ may be the dominant ion depending upon the plasma conditions. Additionally, a multitude of ions with significant intensity are often detected in mixtures of gases, as will be discussed later.

**Fig. 6:**
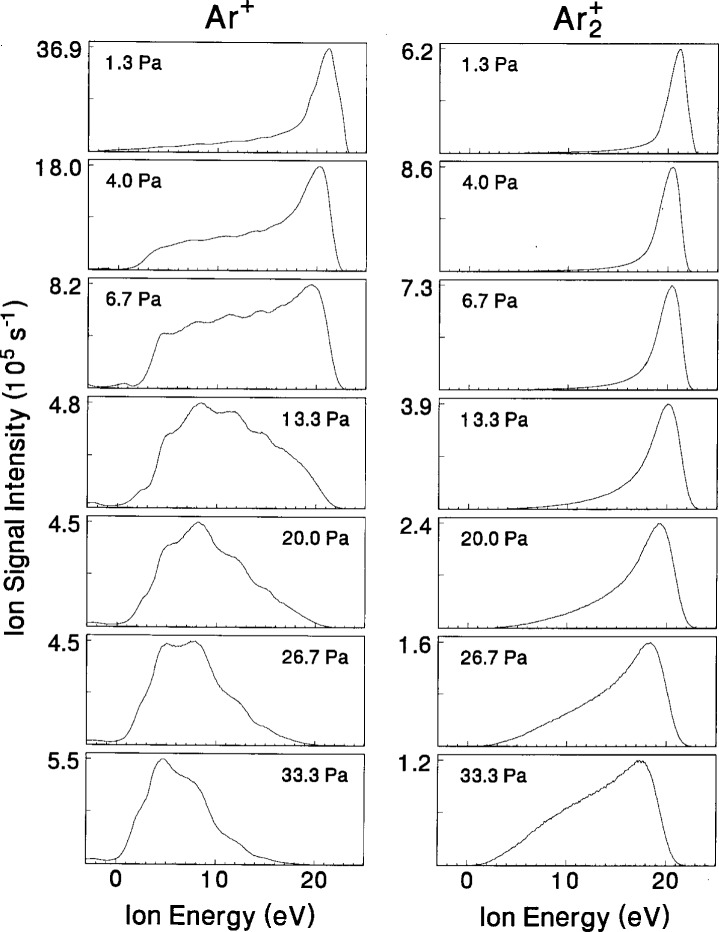
Ion-energy distributions of Ar^+^ and Ar_2_^+^ from argon plasmas with *V*_pp_ = 200 V for pressures ranging from 1.3 Pa to 33.3 Pa (10 mTorr to 250 mTorr) obtained with the EQP system through an orifice in an aluminum electrode. The data are originally from Ref. [21].

While the energy-integrated intensity of the ion signals provides information about the dominant ions, interpretation of the shapes of the IEDs often provide information about the relative roles of different ion-formation processes, e.g., ion-molecule reactions in the sheath and bulk regions of the discharge. Initial investigations of this type in the GEC Cell [20,21] were performed using argon, which had been studied extensively in other rf plasma reactors, and for which the collision mechanisms are reasonably well understood. Similar studies have been performed for N_2_, O_2_, He [22], and H_2_ [23]. Examples of results for argon plasmas are shown by IEDs for Ar^+^ and Ar_2_^+^ in Fig. 6, as a function of pressure.

At 1.3 Pa, the distribution for Ar^+^ is peaked near 21 eV, which is indicative of the magnitude of the time-averaged plasma potential [4]. This is because, under these conditions, most ions are formed in the glow (bulk) region of the discharge by electron-impact ionization, and then travel from the bulk of the plasma to the electrode without experiencing collisions. Thus the ions gain kinetic energy corresponding to the average potential difference between the plasma glow and the potential of the surface of the grounded electrode (*V*_p_ in Fig. 5). As the pressure increases, Ar - Ar^+^ symmetric charge-exchange collisions in the sheath region [16] occur, causing the IED to broaden and the mean kinetic energy to shift toward 0 eV. The secondary peaks or maxima that are observed in the IEDs as the pressure increases are due to the formation of low-energy (thermal) ions within the time-varying rf sheath [16]. In general, the observation of a broadened IED with secondary maxima is an indication of the formation of ions within the sheath region at thermal energies, in most cases predominately the result of charge-exchange collisions.

For comparison, the IEDs for the Ar_2_^+^ ion are also shown in Fig. 6. Like Ar^+^, the Ar_2_^+^ IED is peaked near 21 eV at 1.3 Pa, of the time-averaged plasma potential. However, as the pressure increases, the IEDs for Ar_2_^+^ experience relatively minor broadening and remain peaked near 20 eV for all pressures considered here. This is a clear indication that the observed Ar_2_^+^ ions experience few collisions resulting in a significant loss of kinetic energy as they travel through the sheath. Further, the lack of secondary maxima in the IEDs at higher pressures suggest that these ions are formed only in the bulk region. Minor ions such as Ar_2_^+^, which are formed in the bulk plasma and do not experience significant losses of energy while crossing the sheath, are often useful for determining plasma potentials over a wide range of plasma conditions [4].

The maximum kinetic energies observed in Fig. 6 are all near 20 eV, which is quite low compared to the applied peak-to-peak rf voltage of 200 V, and to ion energies often observed in plasma cells other than the GEC Cell [3,4,7,9,11,14,38]. The low ion energies detected at the grounded electrode are the result of the electrical asymmetry of the GEC Cell, i.e., the powered electrode is much smaller than the “effective grounded electrode” which consists of the grounded electrode and the grounded vacuum chamber [39,40]. This electrical asymmetry is also reflected in the large self-bias voltages that are often observed for the voltage waveforms measured at the powered electrode [29,31].

Studies inferring the role of ion-molecule collisions in the plasma have also been performed for a variety of gas mixtures, where changes in the intensity and energy of ions with changing gas concentrations provide information concerning the dominant collisional interactions within the plasma. The initial study of this type performed on the GEC Cell was for mixtures of argon and helium [24], which were chosen because of the limited number of ion-molecule reactions that were possible within the plasma, and because the collisional cross sections for the gases were reasonably well known. As expected, it was found that, with the exception of small fluxes of the ion ArHe^+^, the ion species did not change with the relative concentrations of the gases. However, it was determined that the fluxes of ions (primarily Ar^+^ and He^+^) increased with increasing helium concentration, which suggested that various ionization processes involving metastable singlet and triplet states of helium are important in these discharges.

A similar study was performed for a gas mixture of argon and hydrogen [25]. This mixture is of interest because of its use in plasma cleaning processes of silicon surfaces [41]. In contrast to other gases, the ion flux in this mixture was not dominated by a single ion. Depending upon the pressures and concentrations studied, as many as five different ions (Ar^+^, ArH^+^, H^+^, H_2_^+^, and H_3_^+^) were observed to have nearly comparable intensities. The relative concentrations of the ions as a function of pressure, along with known collisional cross sections [42,43] were then used to identify the dominant ion-molecule reactions in the Ar-H_2_ plasmas. Additionally, the role of these ions as precursors to the formation of fast H atoms was investigated.

Recent work [26] to determine IEDs from a plasma in a 50/50 volume fraction mixture of He and N_2_ in the GEC cell showed that N_2_^+^ is the dominant ion with minor contributions from N^+^, N_3_^+^, N_4_^+^, and He^+^. The IEDs for N_2_^+^, N^+^, and He^+^ were in reasonable agreement with IEDs obtained from self-consistent hybrid Monte Carlo-fluid simulations [26], but discrepancies between the measured and calculated IEDs for N_3_^+^ and N_4_^+^ ions indicated the possible significance of collisions in the discharge that destroy these ions. Cross sections for these destruction processes were estimated by fitting calculated IEDs to the measured IEDs.

**Fig. 7:**
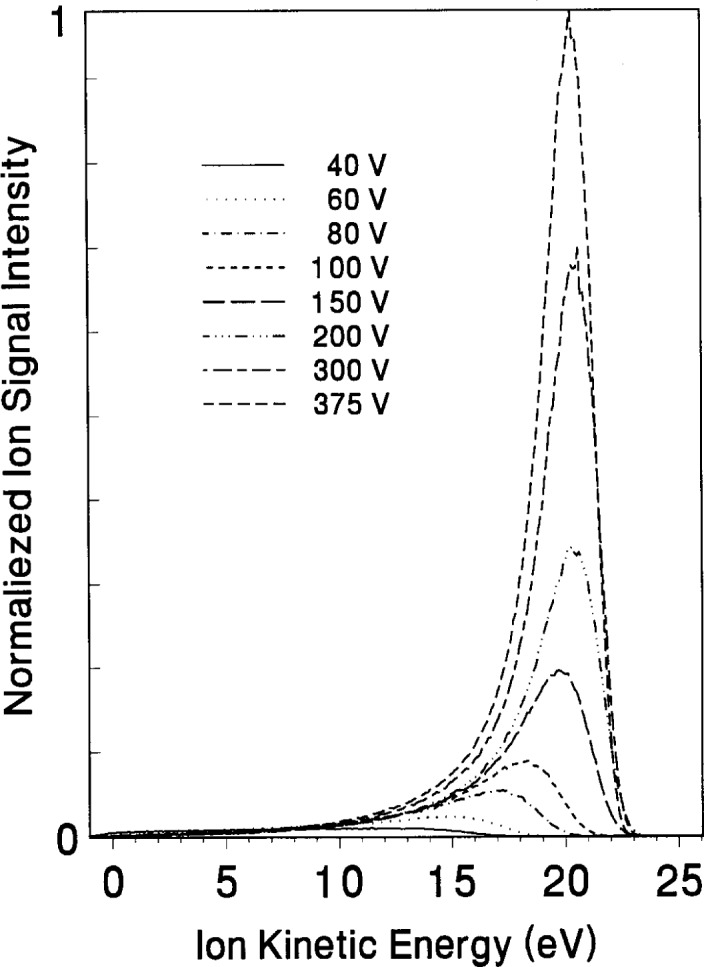
Ion-energy distributions of Ar_2_^+^ ions from argon plasmas at a pressure of 13.3 Pa and applied peak-to-peak rf voltages ranging from 40 V to 375 V. The data were obtained with the EQP system, and the ions were sampled through an aperture in a stainless-steel electrode.

Attempts have also been made to correlate IEDs measured in the GEC Cell with results from measurements made using other diagnostic techniques. One of the more productive efforts involves the correlation of electrical measurements, such as self-bias potentials and applied voltages, to plasma potentials determined from measured IEDs. As mentioned previously, ions such as Ar_2_^+^, that experience little loss of energy as they cross the sheath, are useful in determining the time-averaged plasma potential which is related to the self-bias potential of the plasma. Figure 7 shows the IEDs for Ar_2_^+^ ions at various applied peak-to-peak rf voltages for an argon plasma at 13.3 Pa. It can be seen that the peak energy of the ions increases significantly as *V*_pp_ increases from 40 V to 150 V, but then remains nearly constant up to *V*_pp_ = 375 V. A similar effect is evident in Fig. 6, where the peak ion energy for Ar_2_^+^ is nearly constant with increasing pressure. These trends correlate with the measured self-bias potentials [29] for argon plasmas. Studies of current flow in the cell and the division of voltages across different portions of the discharge have shown that this effect is due to increasing current flow to the chamber walls as the pressure or voltage is increased [44]. Other preliminary studies have been published showing the correlation between various electrical measurements and measured IEDs in plasmas sustained in Ar-O_2_ mixtures [27,45]. These mixtures represent an interesting system because a small amount of oxygen added to an argon rf discharge is observed to produce a significant change in both the measured self-bias potential and corresponding plasma potential, as discussed in the next section.

**Fig. 8:**
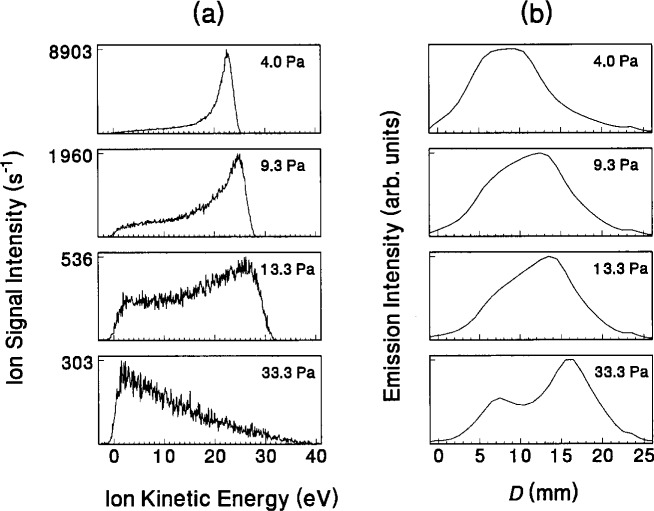
(a) Kinetic-energy distributions of N_3_^+^ ions sampled from nitrogen rf discharges for pressures ranging from 4.0 Pa to 33.3 Pa with *V*_pp_ = 200 V, using the EQP system and a stainless-steel electrode. (b) Vertical optical emission (337.1 nm) profiles for the same conditions as in (a). *D* is the distance of the observation point for the optical measurement from the grounded electrode.

Correlations have also been made between IEDs and the measured optical emission from the discharge. One example is the investigation of the correlations between Doppler-broadened optical emission from fast H atoms and the ion flux present at the grounded electrode [25]. More recently, correlations between optical emission profiles (relative intensity of emission as a function of position between the parallel plates) and the measured IEDs have been attempted. An example of this is shown in Fig. 8 where IEDs for N_3_^+^ ions and optical emission profiles are shown for nitrogen plasmas over a range of pressures with *V*_pp_ = 200 V. The maximum ion energies (which are related to the time-averaged voltage drop across the sheath in front of the grounded electrode) are observed to increase from 25 eV to 40 eV with increasing pressure, while the measured self-bias voltages exhibit a corresponding trend toward smaller absolute magnitudes [22]. This implies that the voltage drop across the ground sheath is increasing with increasing pressure, which means that the plasma is becoming more symmetric. This shift in degree of symmetry can also be observed in the optical emission data in column (b) of Fig. 8. At 4.0 Pa, the sheath in front of the powered electrode (as defined as the width of the dark region) is significantly larger than in front of the grounded electrode. However, as the pressure increases, the sheaths become comparable in size, suggesting similar sheath potentials. The corresponding emission profile exhibits the double bands of brightness that have been previously observed by Bletzinger and De Joseph [46].

## 4. IEDs from Argon-Oxygen Plasmas

In this section we present measured kinetic-energy distributions for ions striking the grounded electrode of the GEC Cell for rf discharges sustained in mixtures of argon and oxygen over a range of oxygen volume fractions from 0 % to 14 %. The argon-oxygen system is of interest because previous measurements have indicated the presence of significant effects with the addition of oxygen to argon plasmas [27,45], and because of the industrial uses of Ar-O_2_ plasmas, that include photoresist stripping [47] and surface cleaning [48]. The data and analysis presented here are meant to serve as an example of the methodology for the experiments discussed in the previous section.

**Fig. 9:**
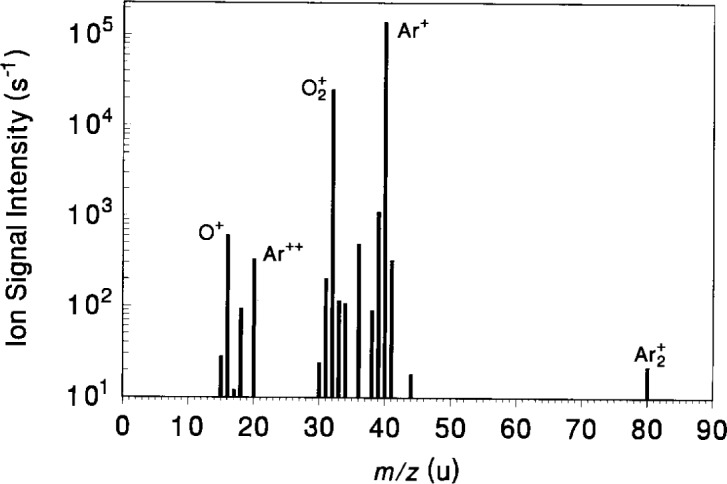
Mass spectrum of ions sampled from a discharge in the GEC Cell sustained in a mixture of argon with 14 % oxygen volume fraction. The gas pressure was 13.3 Pa and *V*_pp_ = 200 V. The ion-energy analyzer was set to transmit ions with 15 eV of kinetic energy.

The identity of ions generated within the plasma is determined by observing mass spectra of the ions entering the sampling orifice over the range of plasma conditions to be studied. Figure 9 shows a sample mass spectrum of the ions generated in an Ar-O_2_ plasma (14 % volume fraction of O_2_) at 13.3 Pa with *V*_pp_ = 200 V. Because the ions must pass through the energy analyzer, only ions with a specific kinetic energy (15 eV in Fig. 9) can be detected in this mode of operation. Therefore, data such as shown in Fig. 9 cannot be used to determine the relative net fluxes of the ions striking the electrode. The IEDs for each ion must be integrated over their entire energy range in order to estimate relative ion fluxes, as discussed below.

From Fig. 9, it is apparent that the dominant ions (at this kinetic energy and relative volume fraction) are Ar^+^ and O_2_^+^, with less abundant ions of interest also present, such as Ar^++^, O^+^, and Ar_2_^+^. Other peaks in the mass spectrum are due to impurities in the vacuum system (such as H_2_O) or isotopes of the gases (such as ^36^Ar). No ions are observed that result from chemical interactions between the two gases in the mixture, in contrast to the formation of ArH^+^ in Ar-H_2_ mixtures [25] and ArHe^+^ in mixtures of argon and helium [24]. The IEDs for Ar^+^, Ar^++^, Ar_2_^+^, O_2_^+^, and O^+^ were obtained for plasmas in Ar-O_2_ mixtures with oxygen volume fractions ranging from 0 % to 14 % at a total pressure of 13.3 Pa. The IEDs presented here were obtained using both the EQP and CMA/MS measurement systems. The simultaneously determined electrical characteristics of the discharge are also reported.

**Fig. 10:**
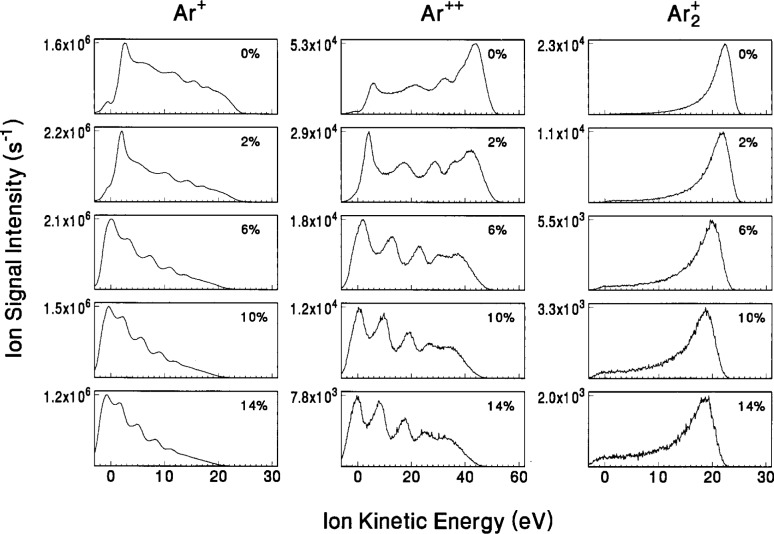
IEDs of “argon ions” sampled from rf discharges in mixtures of argon and oxygen at the indicated volume fractions of O_2_. The ions were sampled through a clean stainless-steel grounded electrode and were analyzed using the EQP apparatus. The gas pressure was held constant at 13.3 Pa, and *V*_pp_ = 200 V.

**Fig. 11:**
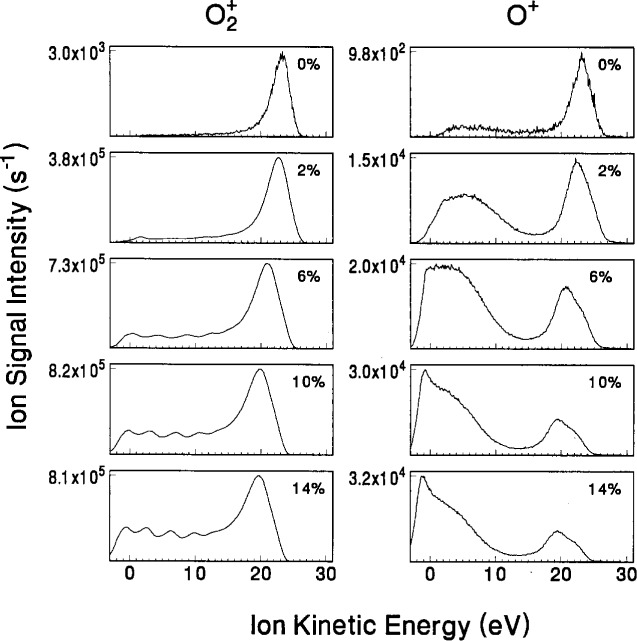
IEDs of “oxygen ions” sampled from rf discharges in mixtures of argon and oxygen at the indicated volume fractions of O_2_. The ions were sampled through a clean stainless-steel grounded electrode and were analyzed using the EQP apparatus. The gas pressure was held constant at 13.3 Pa, and *V*_pp_ = 200 V.

In Figs. 10 and 11, the IEDs for ions resulting from the argon and oxygen gases, respectively, are presented as measured by the EQP apparatus while sampling ions through an orifice in a clean stainless-steel electrode. The IEDs in Figs. 10 and 11 were all measured with the same EQP settings, so the magnitude of the signals may be used to estimate relative fluxes of ions, within the limitations of the mass-dependent ion transmission and detection of the quadrupole mass spectrometer. The IEDs in Fig. 10 for 100 % argon are similar to those previously published [21] for pure argon, except that the sensitivity to low-energy ions is improved due to the use of the clean stainless-steel electrode [33]. The IEDs for Ar^+^ and Ar^++^ are broad and exhibit the secondary structure that is indicative of ion formation in the sheath by charge-exchange collisions [16,49]. By contrast the Ar_2_^+^ ion exhibits the single peak in the IED that is expected for ions that are formed only in the bulk plasma, and experience few collisions in the sheath that result in the loss of kinetic energy. Formation of dimer ions, such as Ar_2_^+^, are thought to be due to three-body collisions [50] or ionizing collisions between highly-excited and ground state argon atoms [51]. As the oxygen concentration increases, the shapes of the IEDs become more heavily weighted toward lower energies for all of the ions, although the effect is greatest for Ar^+^ and Ar^++^. This effect is most probably due to the increasing width of the sheath in front of the grounded electrode that can be observed visually as oxygen is added to the plasma, resulting in a longer mean ion path and a corresponding increase in probability of collisions.

The ion signals for O_2_^+^ and O^+^ for the “0 % oxygen” condition shown in Fig. 11 are the result of ionization of residual oxygen and water molecules that are always present in the vacuum system. The O_2_^+^ IED exhibits a narrow peak similar to Ar_2_^+^, which indicates that no significant energy loss occurs due to collisions between O_2_^+^ and Ar. As the oxygen content increases, the increased probability of symmetric charge-exchange collisions between O_2_^+^ and O_2_ molecules [52] is evident in the appearance of O_2_^+^ ions with lower energies, and the growing presence of secondary structure in the IEDs.

The IEDs for O^+^ exhibit a double peak structure for all concentrations of O_2_, with the peak at lower energies growing in magnitude as the O_2_ content is increased. The peak near 20 eV is due to the formation of O^+^ ions at the bulk-sheath interface by electron-impact dissociative ionization, while the peak at lower energies is most probably due to dissociative ion-molecule reactions in the sheath [53]. Interestingly, the peak at low energies exhibits little or no secondary structure, even though it represents ions formed in the sheath. Beam experiments have shown [54] that dissociative ionization processes for O form O_2_^+^ fragments with significant kinetic energy (several eV). This additional formation energy may be enough to smear out secondary peaks in the IED. The IEDs for O^+^ in pure oxygen discharges do not exhibit a similar peak at low energies [22], suggesting that O^+^ is formed in the argon-oxygen discharges by collisions involving both an argon and oxygen species, or that the formation of O^+^ is significantly affected by changes in the plasma due to the presence of argon. More theoretical work is required to fully understand these complex IEDs.

An important point to note from the IEDs in Figs. 10 and 11 is that the ion signal observed at energies below 0 eV increases as the oxygen content increases in the plasma. This is evidence of the negative energy shift which was discussed in Sec. 2 and shown in Fig. 4. Normally the use of a stainless-steel electrode minimizes this effect, but in this case we observe that the longer the plasma is operated (the experiment began with a clean stainless-steel grounded electrode with 0 % added oxygen and then proceeded sequentially with the higher volume fractions) the more dramatic the shift. This is evidence of the sputtering of aluminum from the powered electrode onto the grounded electrode as discussed in Sec. 2. The process of sputtering aluminum onto the stainless-steel electrode has been observed in this experimental system for many gases, but it is accelerated in this gas mixture, probably due to the large sputtering yield of argon and the oxidation effects of oxygen.

**Fig. 12:**
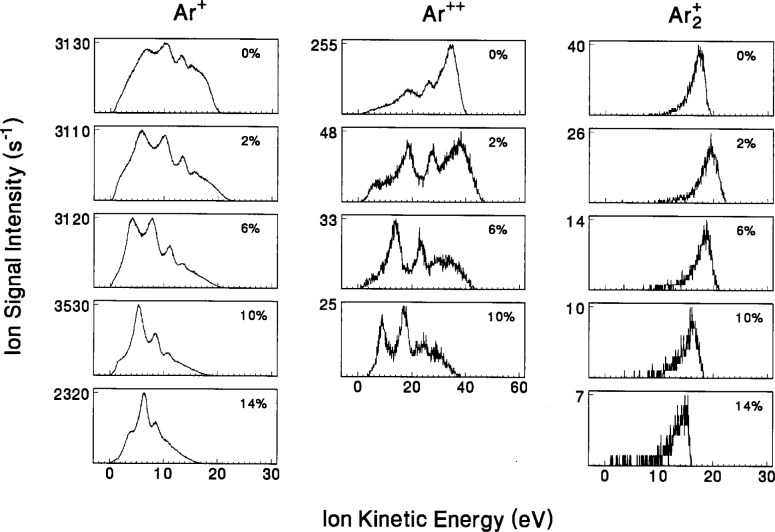
IEDS of “argon ions” sampled from rf discharges in mixtures of argon and oxygen at the indicated volume fractions of O_2_. The ions were sampled through a stainless-steel grounded sampling cone positioned at the edge of the discharge, and were analyzed using the CMA/MS apparatus. The gas pressure was held constant at 13.3 Pa, and *V*_pp_ = 200 V.

**Fig. 13:**
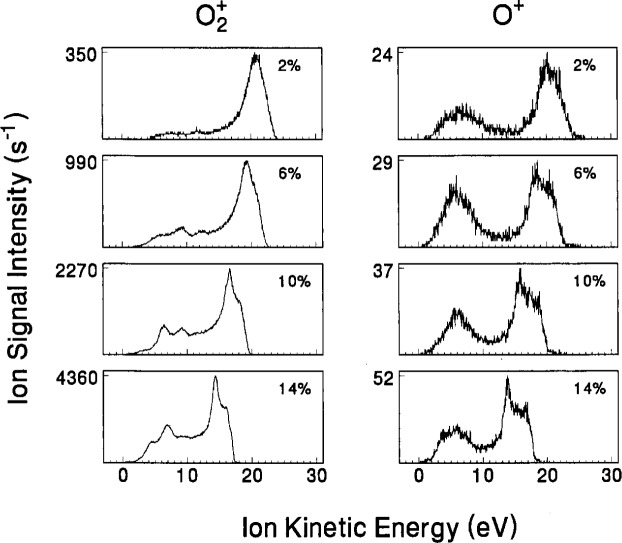
IEDs of “oxygen ions” sampled from rf discharges in mixtures of argon and oxygen at the indicated volume fractions of O_2_. The ions were sampled through a stainless-steel grounded sampling cone positioned at the edge of the discharge, and were analyzed using the CMA/MS apparatus. The gas pressure was held constant at 13.3 Pa, and *V*_pp_ = 200 V.

Presented in Figs. 12 and 13 are data taken using the CMA/MS system shown in Fig. 1, under ostensibly identical plasma conditions as those in Figs. 10 and 11. These data were presented previously along with preliminary analysis [27], and are presented here to provide a more complete comparison between the different experimental arrangements. The IEDs obtained with the CMA/MS system all exhibited an apparent energy shift of approximately +3.5 eV, and the energy scales of the IEDs shown in Figs. 12 and 13 have been adjusted to account for this, as described in Sec. 2 and in Ref. [20]. As for the EQP data, the same CMA/MS settings were maintained for all ions and plasma conditions in order to allow an estimate of relative ion intensities.

The IEDs in Figs. 12 and 13 exhibit the same general trends and features that were discussed above for Figs. 10 and 11. Obvious differences include low-energy discrimination and lower overall sensitivity due to the lack of an extraction field, and in some cases more clearly defined secondary structure due to better energy resolution (see Table 1). In general, the observations and general conclusions drawn in the previous paragraphs from Figs. 10 and 11, are applicable to both sets of IEDs. However, since the IEDs obtained with the EQP configuration do not suffer from significant low-energy discrimination, the shape of the distributions in Figs. 10 and 11 more accurately reflect the distribution of energies of ions actually striking the electrode, and therefore are more appropriate for comparison with theoretical calculations and predictions [10].

**Fig. 14:**
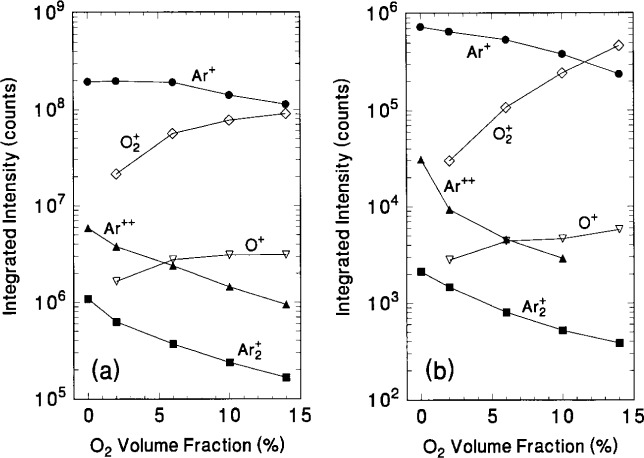
Integrated ion intensities as a function of oxygen volume fraction as derived from the IEDs shown in Figs. 10–13. Data are presented for (a) the EQP system and (b) the CMA/MS system.

The IEDs in Figs. 10 and 11 were obtained using the same setting on the EQP for each mass and plasma condition, so integration of the measured intensities may be used to estimate the relative fluxes of the plasma ions. The same is true for the IEDs obtained with the CMA/MS shown in Figs. 12 and 13. Results of the integration of the IEDs presented in Figs. 10–13 for the EQP and CMA/MS systems are shown in Figs. 14a and 14b, respectively. The general magnitudes of the integrated intensities (*y*-axis labels) for each graph in Fig. 14 are determined by the sensitivity of each apparatus, and can be affected by the settings of the instrument parameters. A determination of *absolute* ion flux from the measured signal intensities is very difficult, depending upon acceptance angles, transmission efficiencies, detector efficiencies, and other factors, and no attempt has been made to carry out such a calibration for these two systems. Only an estimate of relative intensities of ions as measured for each plasma condition is possible for each of these experimental systems. This analysis is limited by the mass-dependent ion transmission and detection efficiency of the quadrupole mass spectrometer, which is estimated to decrease by approximately a factor of 2 over the mass range of 20 u to 100 u for these systems, according to manufacturer reports.

The trends in relative ion intensities obtained by the two methods agree well, which is an indication of the robustness of the techniques and the consistency of the GEC Cell performance, particularly when one considers that the data in Fig. 14a were obtained two years after the data in Fig. 14b. Analysis of the data in Fig. 14 indicate that O_2_^+^ intensity increases in a manner nearly proportional to the increasing concentrations of O_2_, while the Ar^+^ ion flux exhibits a concomitant decrease in intensity. For oxygen volume fractions exceeding about 10 %, the Ar^+^ and O_2_^+^ fluxes are of nearly equal magnitude despite the significantly larger concentration of argon in the gas mixture. At higher levels of oxygen (> 14 %), O_2_^+^ ions most probably dominate the ion flux striking the grounded electrode.

The O^+^ ion also exhibits an increasing signal intensity with increasing O_2_ concentration, but not as great as O_2_^+^, perhaps due to a reduction in the number of high-energy electrons that are required to dissociatively ionize O_2_ in the sheath/bulk interface. A reduction in the relative number of high energy electrons with increasing O_2_ content is also suggested by the corresponding drop in Ar^++^ intensity. The substantial decrease in Ar_2_^+^ intensity may be attributed to an increasing number of destructive ion-molecule collisions in the sheath involving Ar_2_^+^ resulting from the larger sheath widths in front of the grounded electrode observed at increased oxygen concentrations. However, more information is needed about the collisions between Ar_2_^+^ and other molecules to fully understand the influence of oxygen content upon the Ar_2_^+^ ion flux.

**Fig. 15:**
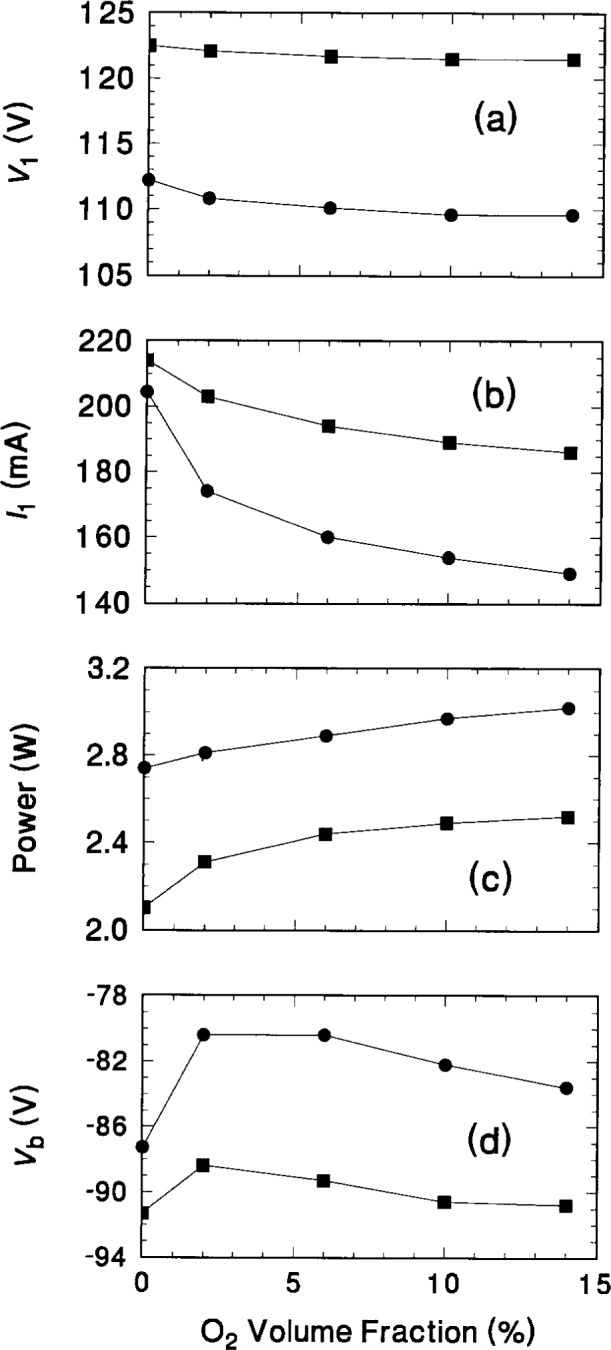
Electrical measurements obtained for Ar-O_2_ plasmas with varying volume fractions of oxygen. Data from the plasmas investigated by both the EQP (squares) and the CMA/MS (circles) systems are presented. *V*_1_ and *I*_1_ are the magnitudes of the first Fourier components of the voltage and current waveforms, respectively, at the surface of the powered electrode, and *V*_b_ is the measured self-bias potential of the powered electrode. The data in (c) is the power dissipated in the plasma, as calculated from the current and voltage waveforms.

Analysis of the electrical measurements obtained simultaneously with the IEDs in Figs. 10–13 is provided in Fig. 15 for both the EQP system (squares) and the CMA/MS apparatus (circles) as a function of oxygen volume fraction. The observed differences between the measurements in Fig. 15 are primarily due to minor changes in the performance of the GEC Cell over time. Additionally, the use of different rf power supplies and digital oscilloscopes for the two experiments made it difficult to accurately reproduce the same magnitude of the applied rf voltage. This is particularly evident in the different values of *V*_1_ (the fundamental Fourier component of the voltage waveform at the surface of the powered electrode), and will result in differing magnitudes of the other measured electrical parameters in the figure. However, experience has shown that the shapes and intensities of the IEDs measured at the grounded electrode are not particularly sensitive to minor changes in the measured electrical parameters. Most importantly, the trends in the electrical measurements are nearly the same for both sets of experimental data.

**Fig. 16:**
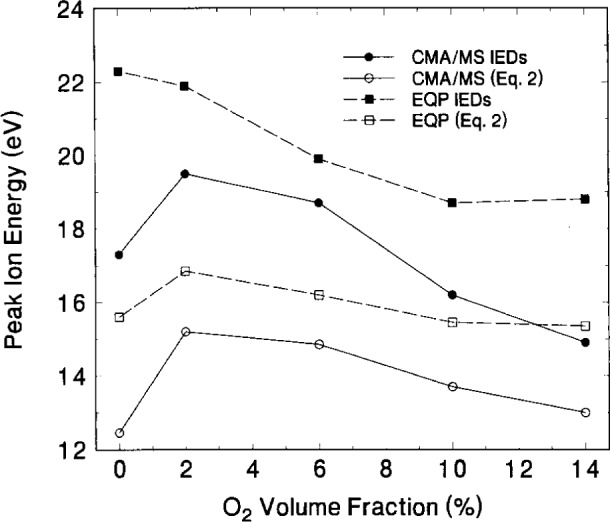
Comparison of time-averaged plasma potentials calculated from Eq. (2) (open symbols) and from Ar_2_^+^ IEDs presented in Figs. 10 and 12 (closed symbols). Data are presented for both the CMA/MS system (circles) and the EQP system (squares).

Kohler et al. [4] have shown that the time-averaged plasma potential as determined from the measured IEDs can be roughly approximated from the electrical measurements of the plasma by

$$(V_{1}+V_{b})/2,$$ (2)

where *V*_b_ is the measured self-bias potential. Calculation of this value for each condition of each data set shown in Fig. 13 provides the open symbol curves in Fig. 16. As seen in Fig. 15, both sets of data exhibit similar trends—the calculated plasma potential rises with the addition of a small amount of oxygen, and then decreases with further additions of O_2_. Comparison of these values with time-averaged plasma potentials (filled symbols) determined from the ion energies in Figs. 10 and 12 corresponding to the peak intensities of the Ar_2_^+^ IEDs, shows that the magnitude of the values are not in agreement. This is to be expected since Eq. (2) does not take into account the floating potential or resistive component of the plasma. However, the trend of the calculated plasma potentials (open circles) from Eq. (2), clearly agrees with the trend in the measured plasma potentials (closed circles) using the IEDs measured by the CMA/MS apparatus in Fig. 12.

The plasma potentials obtained from the EQP data in Fig. 10 (squares) do not exhibit the increase, with the initial addition of oxygen to the plasma, that was observed for the other data, exhibiting instead a generally decreasing slope as the oxygen content is increased. This can be attributed to a shift in the measured plasma potential relative to ground reference caused by surface charging, as discussed above. This suggests that care must be taken when using this technique to determine plasma potentials so that surface effects do not confound the measurements and introduce an uncertainty in the measurement that is difficult to evaluate. Better agreement between the plasma potentials obtained from the EQP data and from Eq. (2) could be achieved by shifting the energy scale of the IEDs in Fig. 10 so that all of the ion signal appears at positive energy values. As noted previously, this combination of gases represents a case where the competition between surface oxidation and sputtering are important in affecting electrode surface conditions that would permit charging. This apparent difficulty has not been observed for other gases and/or mixtures when using a clean stainless-steel electrode.

The differences in the IEDs as measured by the EQP and CMA/MS apparatus are not fully understood. Each system appears to be influenced by surface conditions in a different manner, which makes it difficult to unambiguously define the absolute plasma potentials from the measured IEDs. It is possible that the surfaces through which the ions are sampled for each apparatus are modified in a distinct manner by the plasma due to their differing orientations and positions in the discharge. Furthermore, the different methods of sampling ions, such as the presence or lack of an extraction voltage, may also affect the way that the IEDs are influenced by surface charging. Further analysis of these questions is necessary.

The changes in the measured plasma potential with increasing oxygen concentration have been attributed to a combination of surface and gas phase effects [45], i.e., the production of negative ions in the bulk plasma, and the oxidation of the aluminum electrodes. Preliminary Langmuir probe measurements [55] indicate that the increase in plasma potential at low oxygen concentrations can be attributed to changing of the surface characteristics by interaction with the oxygen-containing plasma, while the decrease at higher concentrations may be related to an increase in negative ion density in the bulk plasma.

## 5. Summary

Kinetic-energy distributions of ions formed in low-pressure discharges generated in the GEC rf Reference Cell have been studied with two different ion energy analyzer-mass spectrometer systems. Both experimental set-ups allow the acquisition of IEDs that provide information about the formation and transport of ions within the discharge. Examples of significant results include the following: (1) verification of electrical behavior of the GEC Cell; (2) identification of the primary ions and ion-collision processes in rf plasmas generated in Ar, He, O_2_, N_2_, H_2_, and related gas mixtures; (3) validation of theoretical models by comparison of measured IEDs with model predictions; and (4) evidence of surface effects upon the measurement of IEDs.

It has been determined that the EQP apparatus allows the measurement of IEDs with improved sensitivity for low-energy ions when sampling through a clean stainless-steel electrode. Sampling through an aluminum electrode or use of the CMA/MS system generates measured IEDs with significant reduction in detected signal at low ion energies. This implies that of the two systems used on the GEC Cell, the EQP system *with a stainless-steel electrode* is more likely to yield measured IEDs that can be compared with theoretical predictions that neglect effects of electrode surface charging. However, analysis of the trends in the IEDs determined by each of the measurement methods provided corroborative information. The IEDs measured with the CMA/MS system did not exhibit energy shifts that were dependent upon the plasma conditions, such as were observed for the EQP apparatus while measuring the IEDs in Ar-O_2_ plasmas. For these experimental conditions, the IEDs obtained with the CMA/MS system were shown to be most suitable for the comparison with electrical measurements.

One of the future challenges for the application of ion energy-mass spectrometry analysis to the GEC Cell include the investigation of IEDs in rf discharges that are generated in gases commonly used for industrial etching processes, such as gases containing chlorine and fluorine. The experimental apparatus and data analysis techniques have been sufficiently well-characterized that the diagnostic technique may now be applied to more complicated gas systems with a reasonable probability of understanding the results. However, the effects of fluorine- and chlorine-containing plasmas on the performance of these experimental systems, including ion sampling, is unknown. Other areas of required investigation include a comparison of measured IEDs between different cells to confirm the reproducibility and reliability of the diagnostic, and the application of the technique to the new inductively coupled plasma source for the GEC Cell [56]. The measurement of IEDs at the powered electrode of the GEC Cell, possibly with a wafer present, and the extension of these techniques to industrial reactors, represent longer term future challenges that need to be addressed.

## Figures and Tables

**Table 1:** Differences between the CMA/MS and EQP apparatus as applied to sampling ions from radio-frequency discharges in the GEC rf Reference Cell

	CMA/MS	EQP
Sampling orifice diameter	0.2 mm	0.1 mm
Sampling location	Side of discharge	Through grounded electrode
Extraction voltage	0 V	−110 V
Type of energy analyzer	CMA	45° ESA
Energy resolution (FWHM)	0.5 eV	1.5 eV

## Footnotes

1 Certain commercial equipment, instruments, or materials are identified in this paper to foster understanding. Such identification does not imply recommendation or endorsement by the National Institute of Standards and Technology, nor does it imply that the materials or equipment identified are necessarily the best available for the purpose.
